# Unrepaired tetralogy of Fallot in a 58-year-old female: factors contributing to the observed survival (case report)

**DOI:** 10.11604/pamj.2023.45.154.29477

**Published:** 2023-08-09

**Authors:** Assia Elouardi, Mohammed Messouak

**Affiliations:** 1 Cardiovascular Surgery Department, Hassan II University Hospital, Fez, Morocco,; 2 Faculty of Medicine and Pharmacy, Sidi Mohammed Ben Abdallah University, Fez, Morocco

**Keywords:** Tetralogy of Fallot, uncorrected, survival, adult congenital heart disease, case report

## Abstract

Tetralogy of Fallot (TOF) is the most common form of cyanotic congenital heart disease. Less than 3% of all patients with uncorrected TOF reach their 40^th^ year of life. We present the case of a 58-year-old woman female with uncorrected TOF. The main factors contributing to her longevity are the early development of aortopulmonary collaterals channels and the relatively gradual narrowing of the right ventricular outflow tract.

## Introduction

Tetralogy of Fallot (TOF) is the most common form of cyanotic congenital heart disease. Most patients undergo radical repair during infancy and childhood. Without surgical intervention, less than 3% of all patients reach the fourth decade of life. We describe the case of a female patient who survived to the age of 58 years with uncorrected TOF.

## Patient and observation

**Patient information:** a 58-year-old nulligravid female patient was admitted to our department with a four-week history of dyspnea New York Heart Association (NYHA) class III dyspnea and cyanosis. Her past medical history included only a cardiac murmur noted in childhood. On further questioning, the patient reported related that she had been diagnosed with TOF since the age of 22. Surgical correction had been suggested, but the patient repeatedly refused treatment.

**Figure 1:**
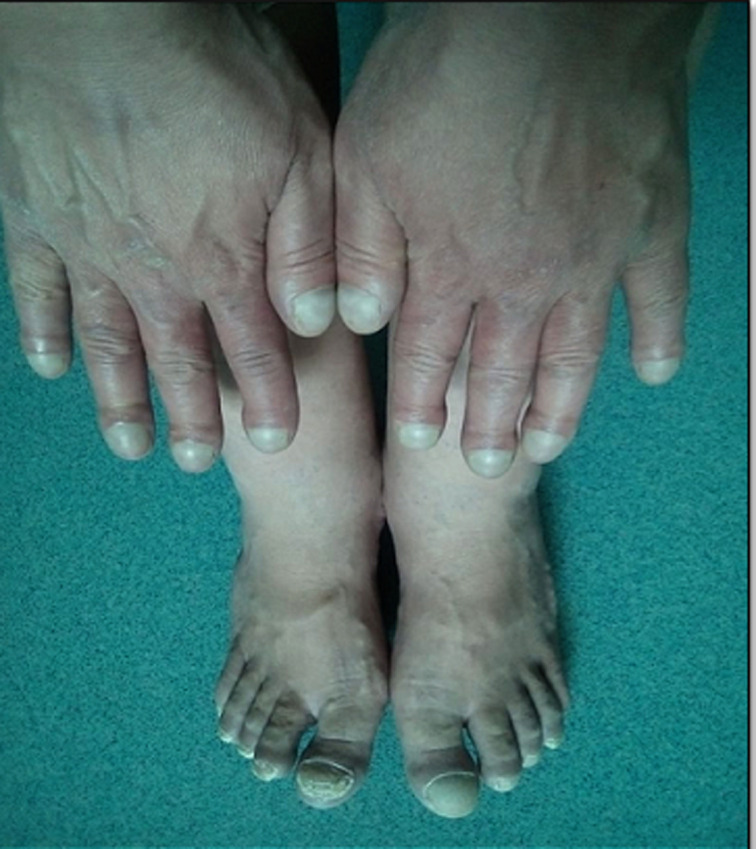
clubbed cyanotic fingers and toes

**Clinical findings:** the physical examination was remarkable for an underdeveloped adult female with oxygen saturation of only 80% on room air. She was 1.56 m tall and weighed 46 kg with a body mass index of 19 kg/m^2^. Her pulse rate was 78 beats/min and her blood pressure was 107/68mmHg. Her lips and oral mucosa were cyanotic. Fingers and toes were clubbed and cyanotic with intact pulses (Figure 1). On cardiac examination, a grade five out of six holosystolic murmur was auscultated over the precordium with a palpable thrill at the apex. Jugular venous pressure was elevated. Pulmonary auscultation revealed clear lung fields. Additionally, hepatomegaly and mild peripheral edema were noted.

**Figure 2:**
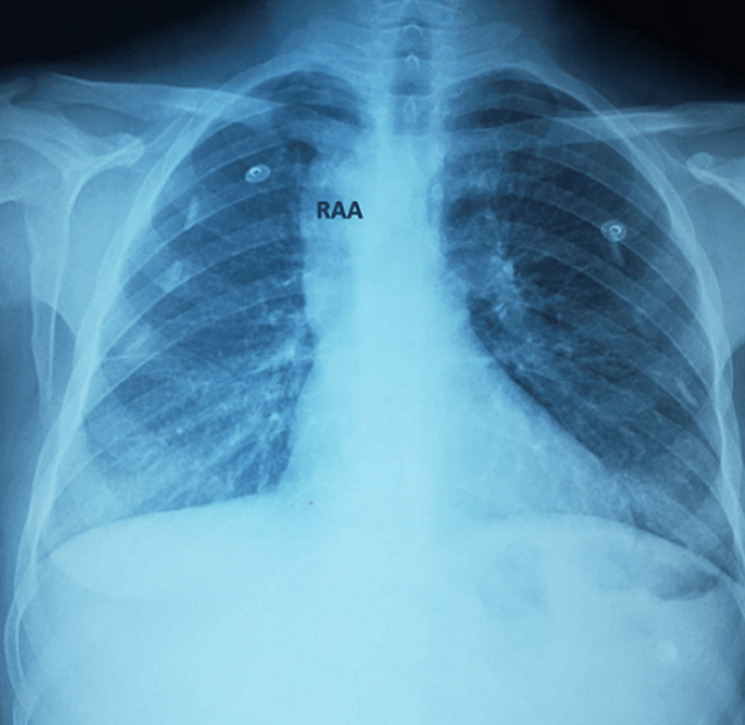
chest X-ray showing right aortic arch

**Figure 3:**
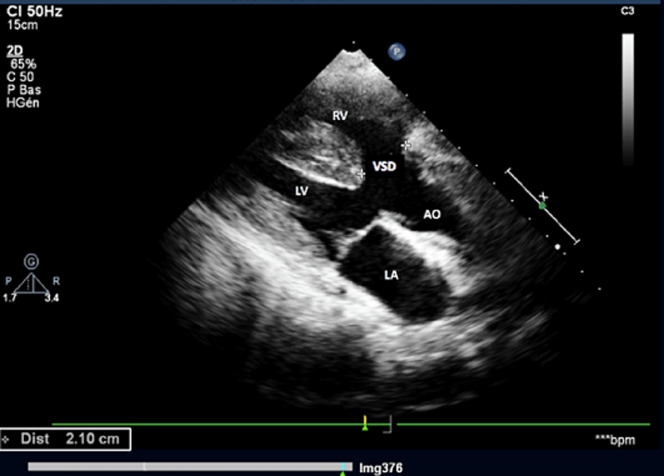
parasternal long-axis echocardiographic view showing ascending aorta overriding the interventricular septum

**Figure 4:**
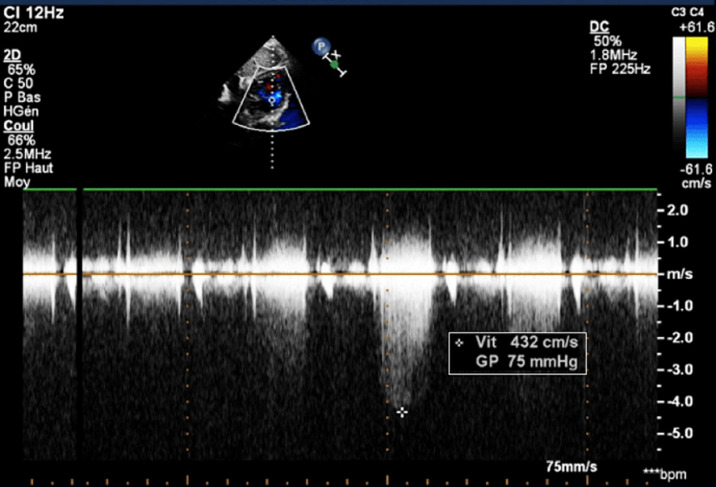
continuous wave Doppler across right ventricular outflow tract in parasternal short-axis view showing a gradient of 75 mmHg

**Figure 5:**
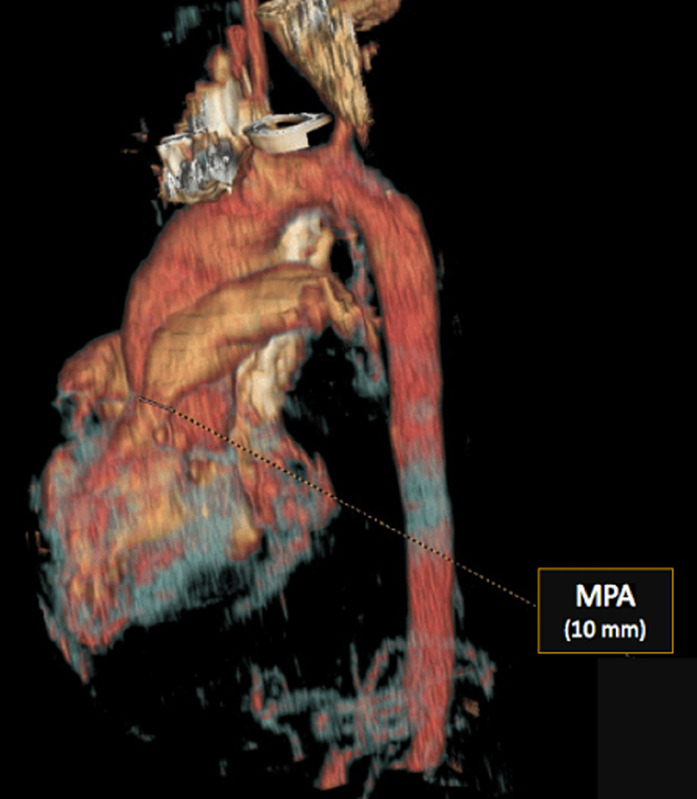
3D contrast-enhanced computed tomography image demonstrating hypoplastic main pulmonary artery

**Figure 6:**
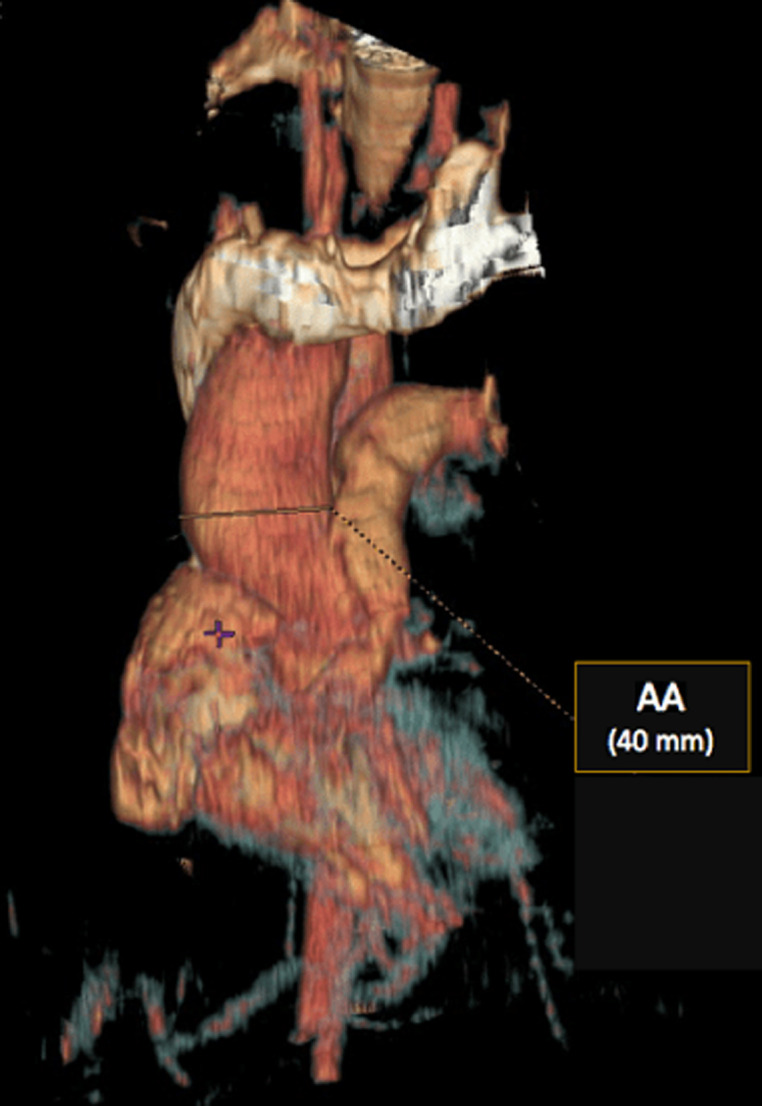
3D contrast-enhanced CT image demonstrating ascending aorta dilation with right aortic arch

**Diagnostic assessment:** the electrocardiogram displayed a regular sinus rhythm of 75 beats per minute, with right axis deviation, voltage criteria consistent with right atrial enlargement and right ventricular hypertrophy (RVH). Chest radiography revealed a right aortic arch (RAA) (Figure 2). Laboratory data included a hemoglobin level of 16.1 g/dl, a hematocrit of 42%, and thrombocytosis, other laboratory data were normal. A two-dimensional echocardiogram revealed moderate right atrial enlargement, right ventricular enlargement and hypertrophy with impaired systolic function, large perimembranous outlet ventricular septal defect (VSD) of 21 mm and an overriding aorta (Figure 3). Doppler examination revealed an infundibular obstruction with a right ventricular outflow tract (RVOT) gradient of 75 mmHg (Figure 4). The main pulmonary artery (MPA) and the right pulmonary artery were mildly hypoplastic compared with the left pulmonary artery. Contrast-enhanced computed tomography (CT) scan confirmed the finding of mild pulmonary artery hypoplasia. The MPA measured 10 mm, the right pulmonary artery measured 9 mm, and the left pulmonary artery measured 20 mm. It also showed RAA, VSD with overriding aorta, RVOT narrowing with RVH and aortopulmonary collateral channels (Figure 5, Figure 6).

**Therapeutic interventions:** after careful consideration of the patient's wishes against corrective surgery and the increased surgical risk associated with the presence of RV dysfunction, the decision was made to manage medically.

**Follow-up and outcome:** the patient was discharged one week later on diuretics and beta-blocker. During a 6-month follow-up, after discharge, she remained in NYHA class II.

**Patient perspective:** the patient was satisfied with the medical management of her condition.

**Informed consent:** written informed consent was granted by patients.

## Discussion

First described in the medical literature by the French physician Etienne-Louis Arthur Fallot in 1888 [1], TOF stands as the most prevalent cyanotic heart disease, accounting for 10-14% of all congenital heart defects [2,3]. The anterocephalic deviation of the outlet septum, with associated abnormal septoparietal trabeculations, is now recognized as the hallmark of tetralogy [4,5]. Classically the TOF is characterized by four primary components: a large ventricular septal defect (VSD), an aorta that overrides both the left and right ventricles, obstruction of the right ventricular outflow tract (RVOT), and compensatory right ventricular hypertrophy (RVH). Additional anomalies may be present with variable frequency, including a right aortic arch (RAA) (25%), atrial septal defect (10%), and coronary artery anomalies (10%) [6]. Childhood cases of TOF typically exhibit cyanosis, hypoxic episodes, failure to thrive, and transient loss of consciousness within the first year of life. In contrast, adult patients with TOF experience progressive dyspnea, worsening cyanosis, and a decline in exercise tolerance. The severity of cyanosis is directly related to the degree of right-to-left shunting. Despite relatively equal right and left ventricular systolic pressures due to the presence of a ventricular septal defect (VSD), persistent shunting occurs as a result of increased pulmonary obstruction [6]. In children, the reported operative mortality rate is below 3% [7]. Early corrective surgery has been observed to facilitate normal growth and development [8]. As a result, early primary repair is considered the preferred treatment approach. Without surgical correction, the number of patients with TOF who reach adulthood is limited, given the average life expectancy of merely 12 years. According to Betranou *et al*. the survival rates without surgical intervention were as follows: 66% at 1 year of age, 40% at 3 years, 11% at 20 years, 6% at 30 years, and 3% at 40 years [9]. Notably, since the first report in 1929 of a patient with uncorrected TOF surviving into late adulthood [10], several papers in the worldwide literature have documented surprising longevity in such cases, with several individuals living into their seventh and eighth decades [11,12].

These findings suggest that our patient is remarkably unique, given her longevity of up to 58 years. This outcome is remarkable, especially considering the predicted survival rate of less than 3% without surgical intervention. The improved survival observed in operated patients may be attributed to anatomic anomalies that are relatively advantageous. Yang *et al*. elucidated three predominant characteristics among operated individuals, which include the presence of a hypoplastic pulmonary artery with a gradual progression of subpulmonary obstruction, as well as left ventricular hypertrophy. Which may act as a balancing factor in the degree of interventricular shunting and could decrease the amount of a right-to-left shunt, or extracardiac shunts including patent ductus arteriosus or systemic-pulmonary artery collaterals for pulmonary blood flow [12]. Our patient did not appear to have left ventricular hypertrophy instead, but rather had early development of aortopulmonary collaterals channels and probably gradual development of RVOT obstruction. For adult TOF repair, the correlation between advanced age and mortality remains controversial in the literature. A study carried out by Hu *et al*. examined 30 patients with TOF, who underwent total repair at the ages of 40 to 60 years revealing an operative mortality rate of 3% [13]. Similarly, in a separate study by Dittrich *et al*. which involved 19 adult patients with TOF who underwent corrective surgery, the observed hospital mortality rate was reported to be 16% [14]. The primary adverse predictors of survival following surgical correction in adult TOF cases include polycythemia, right ventricular (RV) dilatation, advancing age, and elevated RV/LV systolic pressures [15-17]. Adult patients with TOF have a high perioperative mortality rate. Despite this risk, total correction procedures provide clear benefits in terms of improved long-term survival and significantly improved functional status. Similarly, Horer *et al*. documented that 75% of patients achieved a normal lifestyle with full-time employment, while 65.6% were married, and 68.4% of women successfully delivered children [15].

## Conclusion

In our case, after lengthy discussion and deliberation, we made the decision to manage medically, in accordance with the patient's well-informed preferences. The decision was primarily influenced by the current lack of extensive data supporting surgical intervention within this specific age group, particularly when considering the pronounced presence of evident right ventricular dysfunction.
